# Research trends on cancer neuroscience: a bibliometric and visualized analysis

**DOI:** 10.3389/fnins.2024.1408306

**Published:** 2024-08-29

**Authors:** Xinru Ma, Kun Deng, Yingnan Sun, Minghua Wu

**Affiliations:** ^1^The Affiliated Cancer Hospital of Xiangya School of Medicine, Central South University/Hunan Cancer Hospital, Changsha, Hunan, China; ^2^The Key Laboratory of Carcinogenesis of the Chinese Ministry of Health, The Key Laboratory of Carcinogenesis and Cancer Invasion of the Chinese Ministry of Education, Cancer Research Institute, Central South University, Changsha, Hunan, China

**Keywords:** cancer neuroscience, bibliometric analysis, nervous system, VOSviewer, CiteSpace

## Abstract

**Background:**

Recently, cancer neuroscience has become the focus for scientists. Interactions between the nervous system and cancer (both systemic and local) can regulate tumorigenesis, progression, treatment resistance, compromise of anti-cancer immunity, and provocation of tumor-promoting inflammation. We assessed the related research on cancer neuroscience through bibliometric analysis and explored the research status and hotspots from 2020 to 2024.

**Methods:**

Publications on cancer neuroscience retrieved from the Web of Science Core Collection. CiteSpace, VOSviewer, and Scimago Graphica were used to analyze and visualize the result.

**Results:**

A total of 744 publications were retrieved, with an upward trend in the overall number of articles published over the last 5 years. As it has the highest number of publications (*n* = 242) and citations (average 13.63 citations per article), the United States holds an absolute voice in the field of cancer neuroscience. The most productive organizations and journals were Shanghai Jiaotong University (*n* = 24) and Cancers (*n* = 45), respectively. Monje M (H-index = 53), Hondermarck H (H-index = 42), and Amit M (H-index = 39) were the three researchers who have contributed most to the field. From a global perspective, research hotspots in cancer neuroscience comprise nerve/neuron-tumor cell interactions, crosstalk between the nervous system and other components of the tumor microenvironment (such as immune cells), as well as the impact of tumors and tumor therapies on nervous system function.

**Conclusion:**

The United States and European countries are dominating the field of cancer neuroscience, while developing countries such as China are growing rapidly but with limited impact. The next focal point in this field is likely to be neurotrophic factors. Cancer neuroscience is still in its infancy, which means that many of the interactions and mechanisms between the nervous system and cancer are not yet fully understood. Further investigation is necessary to probe the interactions of the nervous system with cancer cell subpopulations and other components of the tumor microenvironment.

## 1 Introduction

**Figure 1:**
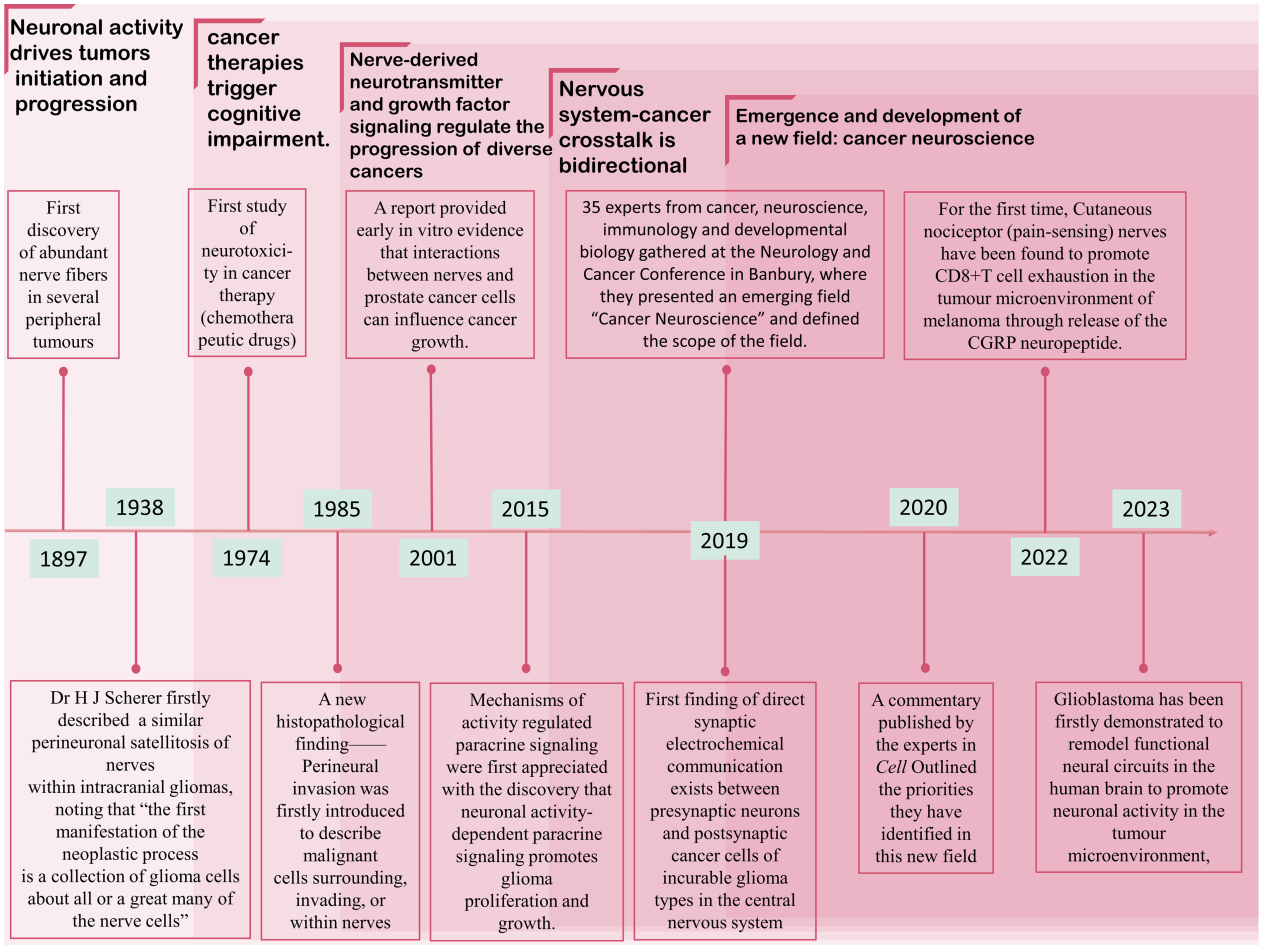
Milestones in cancer neuroscience.

It is becoming more widely acknowledged that the nervous system regulates the development, plasticity, homeostasis, and regeneration of non-neural tissues (Boilly et al., 2017). In particular, an increasing amount of research has revealed that the nervous system plays an indispensable role in cancer pathogenesis and progression (Pan and Winkler, 2022). In turn, tumor progression disrupts and remodels the nervous system to regulate tumor growth (Faulkner et al., 2019). Thanks to these discoveries, a brand-new discipline known as “cancer neuroscience” has emerged [first presented at the Banbury Meeting on the Nervous System and Cancer (10–13 December 2019)], which concentrates on the crosstalk between the nervous system and cancer, both systemic and local (Pan and Winkler, 2022; Mancusi and Monje, 2023). Although “cancer neuroscience” is still in its initial stage, the relationship between cancer and the nervous system has long been studied. Figure 1 illustrated the emergence and development of milestones in the field of cancer neuroscience.

A paper published in the 19th century revealed that nerve fibers were abundant in a number of peripheral tumors (Young, 1897), implying that nerves may play an integral role in malignancy and setting the stage for the field of cancer neuroscience. Recently, both innervation and perineural infiltration (PNI) have been classified as the latest characteristics of the malignant microenvironment (Hanahan, 2022). PNI is the term for the proliferation and invasion of malignant cells along neural fibers, which causes chronic pain syndromes and peripheral nerve remodeling (Wei et al., 2022), and is correlated with aggressiveness and poor prognosis in several cancers, including breast cancer, pancreatic cancer, and prostate cancers (Selvaggi et al., 2022; Villers et al., 1989). Tumors invaded by sympathetic, parasympathetic/vagal, and sensory nerves are referred to as tumor innervation (Cui et al., 2023). The sympathetic and parasympathetic/vagal nerves effect on cancer may be mediated by β-adrenergic receptors or muscarinic receptors and attribute to tumor-associated macrophage polarization, tumor-angiogenesis and changes in cancer cell behavior (Zahalka et al., 2017; Kamiya et al., 2021). To be specific, the sympathetic nerves have a pro-cancer effect, such as in breast and prostate cancer. However, the role of parasympathetic nerves depends on the type of tumor. They have cancer-promoting effects on colorectal, gastric, and prostate cancers, but have cancer-suppressing effects on breast and pancreatic cancers (Zahalka et al., 2017; Kamiya et al., 2021). It has also been demonstrated that sensory nerves contribute to cancer pathogenesis, for example, ablation of sensory neurons can lead to slower growth of pancreatic cancer (Saloman et al., 2016); the invasion and metastatic spread of triple-negative breast cancer can be facilitated by sensory nerve innervation (Le et al., 2016). Moreover, neuronal activity is crucial to the biology of brain tumors, despite the fact that nerve cells and tumor cells interaction has been largely unexplored. For instance, co-culture of glioma cells with neurons resulted in a significant rise in cancer cells (Venkatesh et al., 2015), which was mediated by neuronal activity-regulated paracrine factors and synaptic signaling.

Bibliometrics emerged at the beginning of the 20th century (Hassan et al., 2021). Taking the system of literature and the characteristics of bibliometrics as the research object, it is frequently employed in the quantitative and qualitative analysis of literature (Ninkov et al., 2022). In the process of analysis, it provides access to detailed information on nations, regions, organizations, authors, disciplines, and journals, allowing evaluation of the status and directions of research activities (Priovashini and Mallick, 2022). At present, bibliometrics has become one of the most widely used methods for evaluating the authority and credibility of academic works (Ahmad and Slots, 2021). An up-to-date and comprehensive bibliometric article presenting the current state and future directions of cancer neuroscience is necessary. Therefore, we conducted a bibliometric analysis, aiming to provide research status and prospective direction for subsequent developments in the field by collecting publications from the last 5 years, as well as conducting country, author, journal, citation and keyword analysis.

## 2 Materials and methods

### 2.1 Data collection

**Figure 2:**
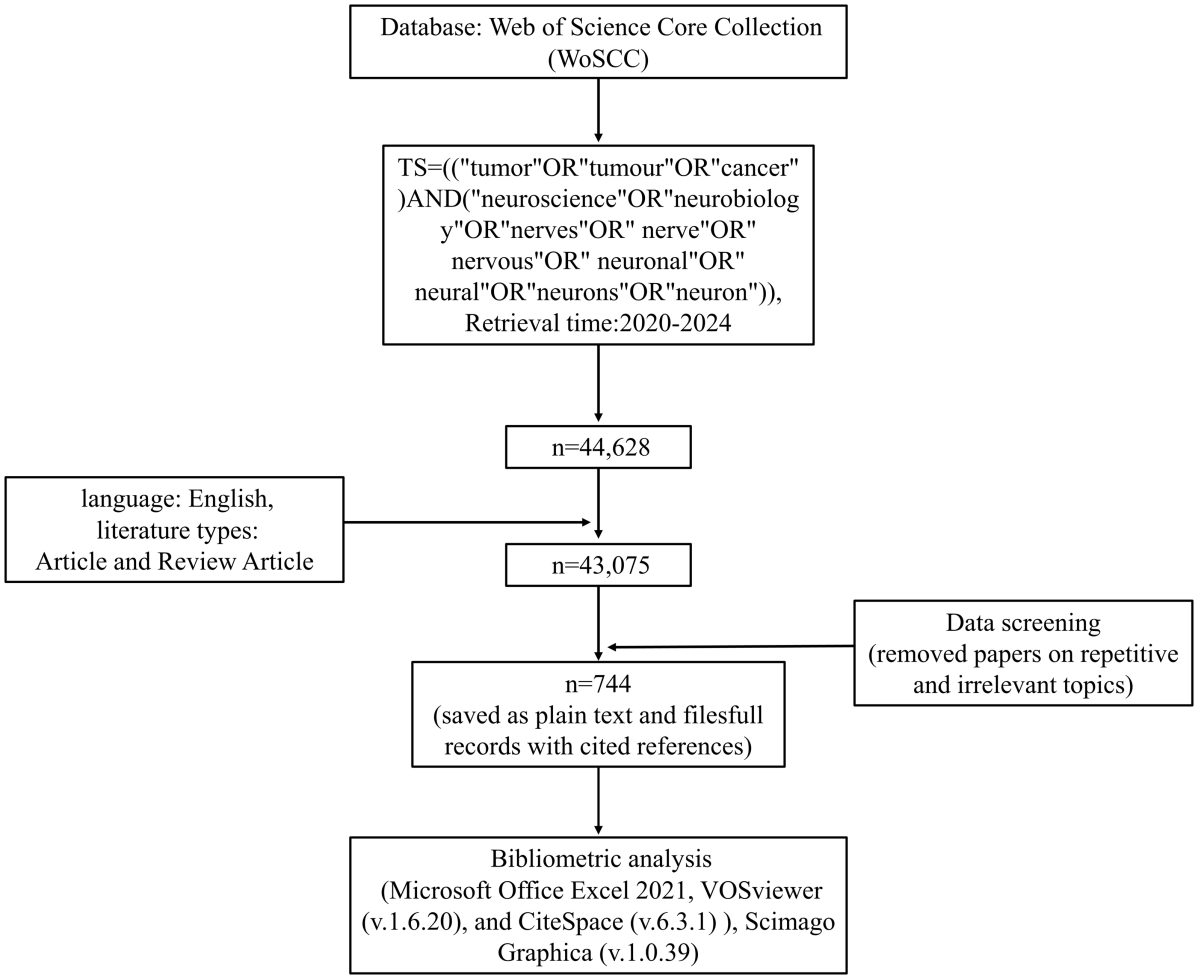
Flowchart of the process of publication selection.

Given that Web of Science covers a wide range of publications in different fields, and can provide a more comprehensive and reliable literature resource than other databases such as Scopus, Medline, and PubMed (Liu et al., 2022; Yeung, 2019), it has been recognized by many researchers as the most suitable database for bibliometric analyses (Wei et al., 2022; Jiang et al., 2023; Wu et al., 2022). Therefore, Web of Science Core Collection (WoSCC) was chosen as the data source for our study. To ensure the comprehensiveness and representativeness of the retrieved data, we designed the search strategy as follows: TS = [(“tumor” OR “tumour” OR “cancer”) AND (“neuroscience” OR “neurobiology” OR “nerves” OR “nerve” OR “nervous” OR “neuronal” OR “neural” OR “neurons” OR “neuron”)], with a time span starting from January 2020 to March 2024. And then, we did some screening: the literature types were restricted to articles and review articles, the language was limited to English, and the papers of duplication and irrelevance to the topic were excluded. Finally, a total of 744 records related to cancer neuroscience, containing cited references, were exported and saved in the download_.txt format as plain text files. The flow diagram of data collection and filtering was shown in Figure 2.

### 2.2 Data analysis

After gathering all reliable data, we used four software to evaluate papers on cancer neuroscience. First, the annual distribution of publications was analyzed by Microsoft Excel 2021. Scimago Graphica was used to visualize publications for each country and the strength of national collaboration.

VOSviewer then further analyzed the data for annual production, author, country, journal, keywords, citations, and more. VOSviewer is bibliometric software with powerful graphical capabilities based on Java, allowing the handling of extensive data (Priovashini and Mallick, 2022). Based on a probabilistic approach to data normalization, it provides multiple visual views of keywords, co-institutions, co-authors, and other fields, including network visualization, overlay visualization, and density visualization (Wei et al., 2022).

CiteSpace is also a bibliometric analysis software that complements VOSviewer. Based on the data normalization method of set theory for measuring the similarity of knowledge units, it provides us with time-zone visualization and timeline visualization and facilitates us to understand the development process and trend of a certain field (Liu et al., 2022; Chen et al., 2022).

## 3 Results

### 3.1 Annual publications on cancer neuroscience

**Figure 3:**
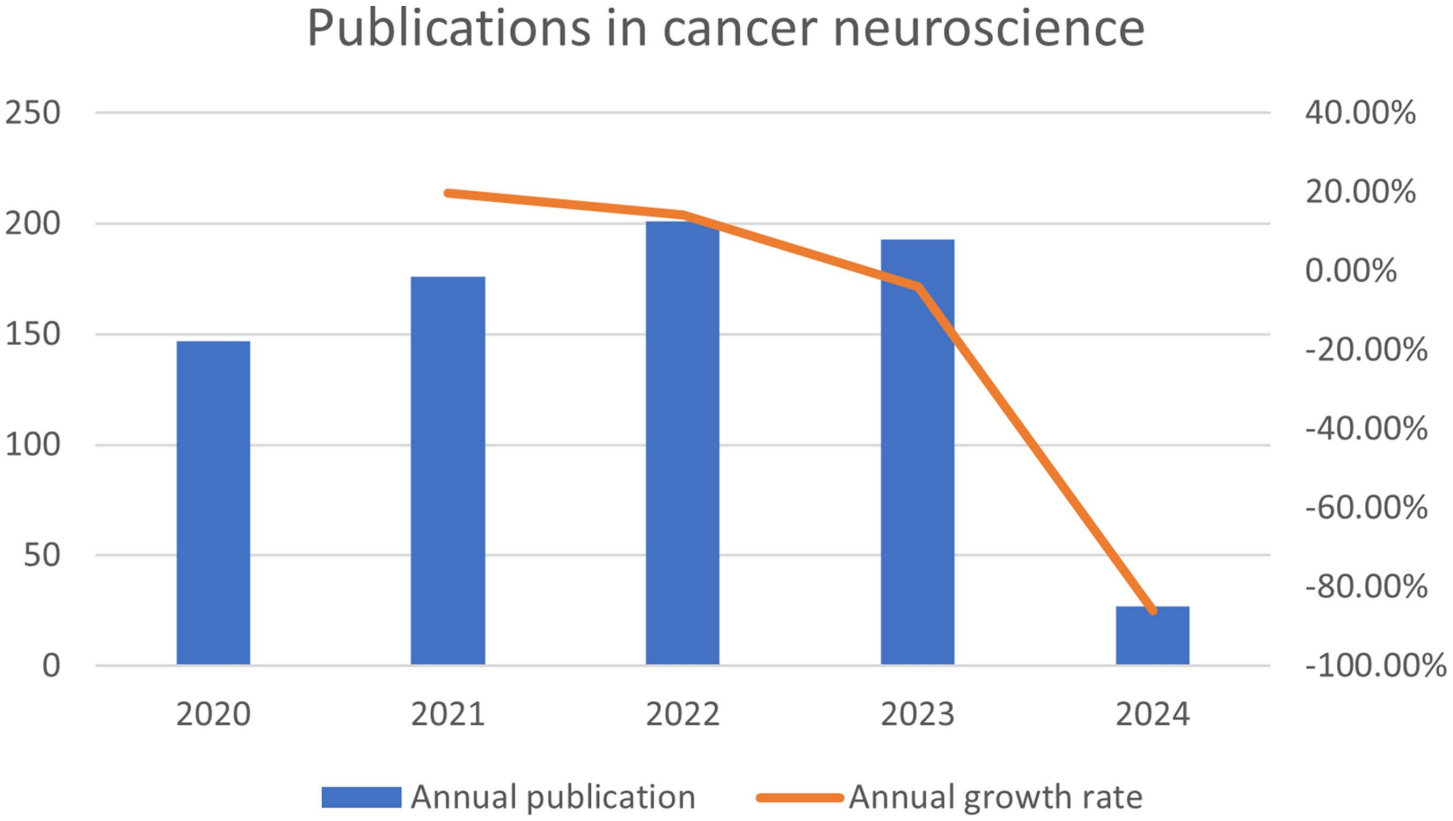
The annual number of publications of cancer neuroscience from January 2020 to March 2024.

In the last 5 years since the concept of cancer neuroscience was introduced, we have obtained a total of 744 documents from the WoSCC contributing to this research field; those 744 documents came from 4,635 authors at 1,169 organizations in 61 countries and were published in 361 journals. Generally speaking, the number of publications has been steadily rising over the past 5 years (Figure 3). From 2020 to 2023, the average number of papers per year was approximately 180. 2021 had the highest output growth rate of 19.7%, indicating a sharp rise in academic attention and study in this field during this period. From 2022 to 2024, although the growth rate of publications in this field has slowed down, researchers or scholars have been paying attention to this new field.

### 3.2 Analysis of countries and organizations

From 2020 to 2024, altogether 61 countries have initiated research in this field. The top 5 countries published publications were performed in Table 1, and the United States and China were far ahead in terms of publications, with 242 and 225 papers, respectively, followed by Italy (Mendenhall et al., 2007), Japan (48 papers), and Germany (46 papers). Considering the average number of citations for published literature in a country, Germany (14.5 citations), the United States (13.63 citations), and Japan (13.46 citations) took the top 3. We can learn that developed countries like the United States and Europe are dominating the field of cancer neuroscience.

**Table 1:** Five most productive countries.

Rank	Countries	Article counts	Average number of citations
1	USA	242	13.63
2	China	225	8.43
3	Italy	51	7.92
4	Japan	48	13.46
5	Germany	46	14.50

**Figure 4:**
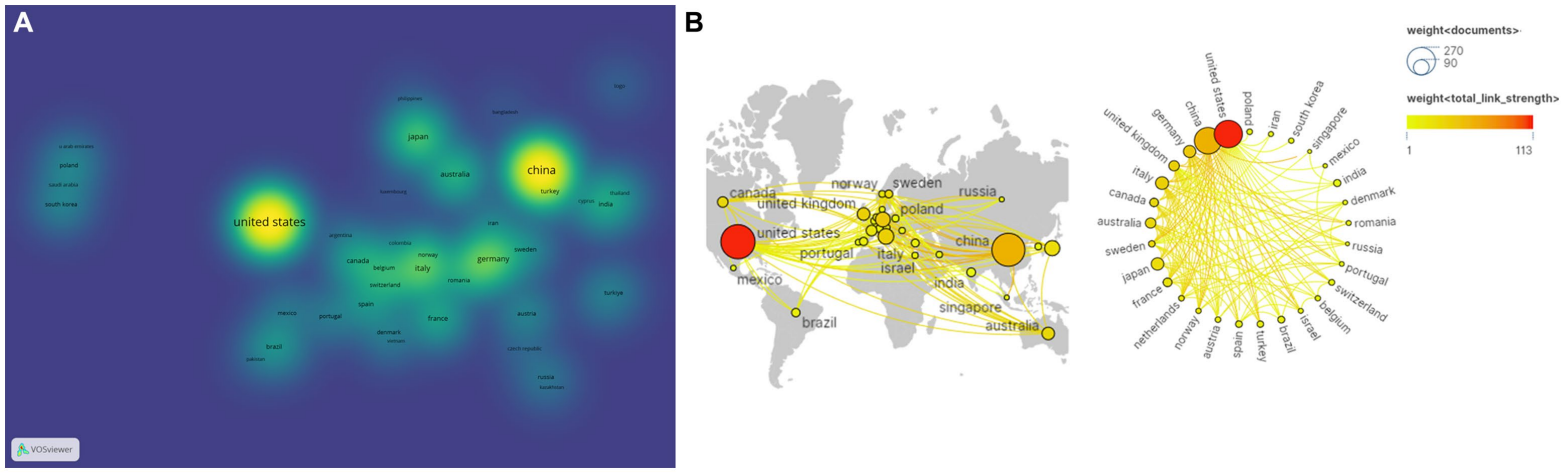
Analysis of country based on the number of publications and the strength of collaboration. **(A)** Publications analysis of countries in density visualization map. **(B)** International collaboration and geographical distribution of country in cancer neuroscience.

And then, the country analysis was visualized by VOSviewer and Scimago Graphica based on publications and the strength of collaboration (Figure 4). Node size reflects the amount of literature published in each country, with larger nodes in the United States and China indicating that these two countries publish more literature (Figure 4A). Figure 4B depicted cooperation between countries, with thicker lines between nodes indicating closer cooperation between countries. Obviously, the United States and China are the central countries in this field and have a close cooperation with Germany, Japan, Italy, and other countries.

Table 2 provided the top 5 organizations with the highest publications. Notably, 3 organizations were from the United States and 2 organizations were from China in the top 5 most productive organizations. Among them, Shanghai Jiaotong University contributed the most research papers (24 papers in total), followed by University Texas MD Anderson Cancer Center (*n* = 22), Stanford University (*n* = 16), University of Pittsburgh (*n* = 13), and Fudan University (*n* = 13). However, the top 3 with the most citations were University Texas MD Anderson Cancer Center (an average of 18.59 citations), Stanford University (an average of 16.31 citations), and Shanghai Jiaotong University (an average of 15.17 citations). Most of them came from the United States, suggesting that research has been conducted primarily in European and American countries, with China producing a large number of publications but still having a limited impact.

**Table 2:** Top 5 organizations based on publications.

Rank	Organizations	Article counts	Average number of citations	Country
1	Shanghai Jiaotong University	24	15.17	China
2	University Texas MD Anderson Cancer Center	22	18.59	USA
3	Stanford University	16	16.31	USA
4	University of Pittsburgh	13	9.38	USA
5	Fudan University	13	7.23	China

### 3.3 Analysis of journals

Next, 361 journals active in the field were analyzed by VOSviewer. Taking 8 as the minimum number of documents for a source, 10 of them satisfied the standards. Based on publications and citations, Table 3 shown the top 10 leading journals in the field.

**Table 3:** The top 10 most active journals in this fields.

Rank	Journal	Article counts	Total number of citations	Average number of citations
1	Cancers	45	332	7.38
2	International Journal of Molecular Sciences	28	266	9.50
3	Frontiers in Oncology	18	144	8.00
4	Cells	16	117	7.31
5	Frontiers in Cell and Developmental Biology	14	127	9.07
6	Advanced Biology	11	53	4.82
7	Nature Communications	10	237	23.70
8	Scientific Reports	10	187	18.70
9	Nature	8	499	61.00
10	BBA Reviews on Cancer	8	83	10.38

The top three journals, depending on publication numbers in recent years, were *Cancers* (*n* = 45), *International Journal of Molecular Sciences* (*n* = 28), and *Frontiers in Oncology* (*n* = 18). It is noteworthy that *Nature*, despite having published only 8 articles in the last 5 years, recorded the highest average citations per article (61.00), which indicated that the quality of the articles published in this journal has received a lot of attention and has a significant impact on cancer neuroscience. In terms of average number of citations, *Nature Communications* (23.70 citations), *Scientific Reports* (18.70 citations), and *BBA Reviews on Cancer* (10.38 citations) were the next most cited journals.

### 3.4 Analysis of authors

In total, 4,635 authors were devoted to cancer neuroscience research. As shown in Table 4, there are 5 highly productive authors with 8 or more publications in the field.

**Table 4:** The top 5 with most published authors in this field.

Rank	Author	Article counts	H-index	Total number of citations	Average number of citations
1	Amit M	12	39	361	30.08
2	Monje M	10	53	170	17.00
3	Hondermarck H	10	42	155	4.20
4	Mravec B	10	23	125	12.50
5	Winkler F	8	7	138	17.25

Among them, Amit M published the most papers, with a total of 12 papers published in the past 5 years (30.08 citations per paper), followed by Monje M (*n* = 10, 17.00 citations per paper), and Hondermarck H (*n* = 10, 4.20 citations per paper). Notably, Amit M (*n* = 12, 30.08 citations per paper), Winkler F (*n* = 8, 17.25 citations per paper), and Monje M (*n* = 10, 17.00 citations per paper) were the most cited authors.

In addition, we introduced another quantitative indicator: the H-index, also known as the number of high citations, which can be used to assess the number of scholarly outputs and the level of scholarly outputs of researchers. Ranked by H-index, Monje M was in the first place as the researcher who has contributed the most to the field (H-index = 53), followed by Hondermarck H (H-index = 42) and Amit M (H-index = 39).

### 3.5 Analysis of citations

**Figure 5:**
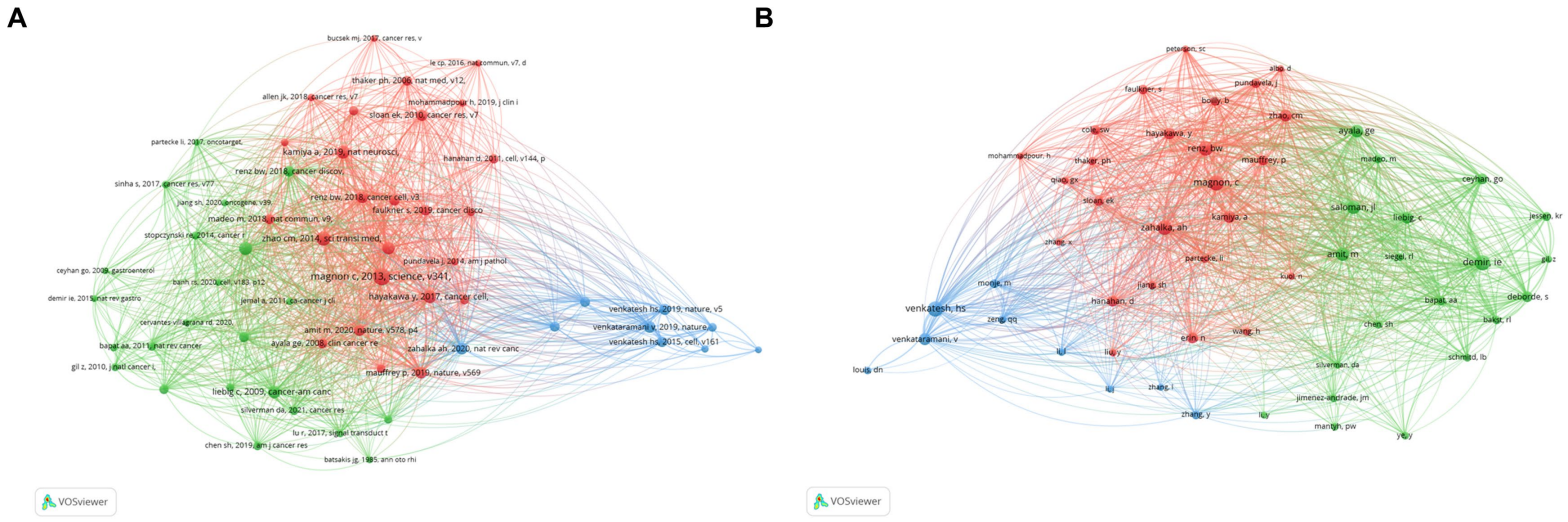
Co-citations analysis. **(A)** Co-citations analysis of cited references. **(B)** Co-citations analysis of cited author.

By choosing 30 as the minimum citation number of a cited reference, the citation network was visualized using VOSviewer. All the cited references were divided into three clusters (Figure 5A). As shown in Figure 5A, the blue cluster focused on synaptic connections of neurons to brain tumors; the cited references in the red cluster focused on how the autonomic nervous system regulates tumor genesis and development via neurotransmitters and neurotrophic factor signaling; and the green cluster focused on the mechanisms of tumor hijacking and remodeling the nervous system to promote invasion and progression.

The top 10 most cited references were reported in Table 5. These are all noteworthy and influential foundational publications on this topic that have a significant impact on future studies. *Autonomic nerve development contributes to prostate cancer progression* (2013; 192 citations), *Denervation suppresses gastric tumorigenesis* (2014; 130 citations), and *Genetic* manipulation *of autonomic nerve fiber innervation and activity and its effect on breast cancer progression* (2019; 106 citations) were the top three cited references.

**Table 5:** Top 10 cited references.

Rank	Title	Author	Year	Journal	Citation
1	Autonomic nerve development contributes to prostate cancer progression	Magnon C	2013	Science	192
2	Denervation suppresses gastric tumorigenesis	Zhao CM	2014	Science Translational Medicine	130
3	Genetic manipulation of autonomic nerve fiber innervation and activity and its effect on breast cancer progression	Kamiya A	2019	Nature Neuroscience	106
4	Nerve Growth Factor Promotes Gastric Tumorigenesis through Aberrant Cholinergic Signaling	Hayakawa Y	2017	Cancer Cell	105
5	Ablation of sensory neurons in a genetic model of pancreatic ductal adenocarcinoma slows initiation and progression of cancer	Saloman JL	2016	Proceedings of the National Academy of Sciences of the United States of America	103
6	Perineural invasion in cancer: a review of the literature	Liebig C	2009	Cancer	101
7	Adrenergic nerves activate an angio-metabolic switch in prostate cancer	Zahalka AH	2017	Science	95
8	Nerves in cancer	Zahalka AH	2020	Nature Reviews Cancer	92
9	β2 Adrenergic-Neurotrophin Feedforward Loop Promotes Pancreatic Cancer	Renz BW	2018	Cancer Cell	90
10	Progenitors from the central nervous system drive neurogenesis in cancer	Mauffrey P	2019	Nature	88

Moreover, we set the minimum number of citations for cited authors to 45 and generated a co-citation network of cited authors by VOSviewer. As shown in Figure 5B, the cited authors in the red cohort worked on drug management or Schwann cells and tumor microenvironment; those in the green cohort worked on the crosstalk of nervous system-immune system-cancer; and those in the blue cohort worked on neuronal-cancer interactions and glioma integration with neural networks.

### 3.6 Analysis of keywords

Keywords are a distillation and summary of what is being expressed in a paper. Through keyword analysis, we can identify trends and research hotspots in related fields.

**Figure 6:**
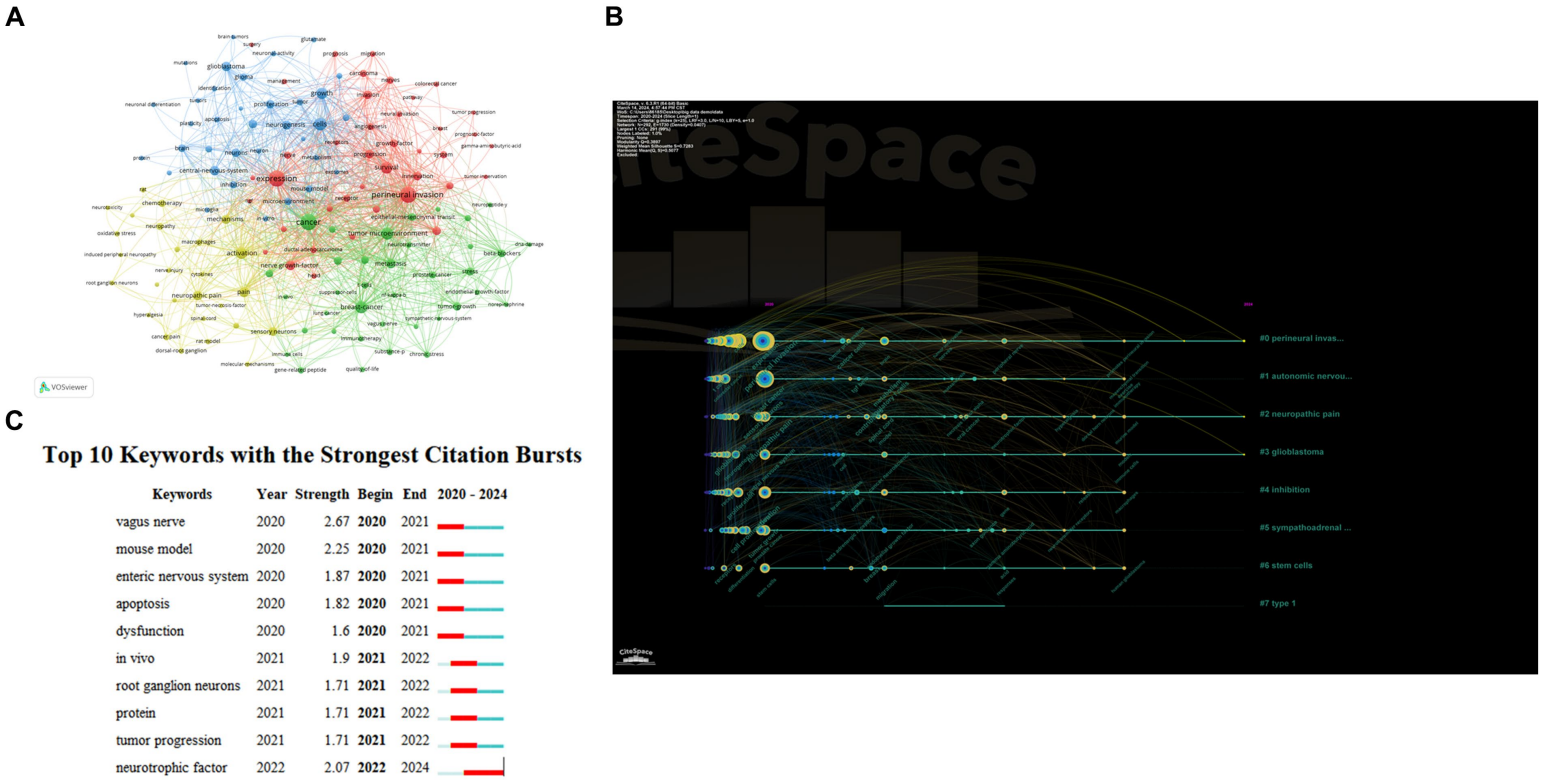
Analysis of keywords. **(A)** Co-occurrence analysis of keywords. **(B)** The timeline view for keywords. **(C)** Top 10 Keywords with the Strongest Citation Bursts.

After selecting the occurrence frequency to be more than 10 VOSviewer divided keywords into four clusters (Figure 6A). In the red cluster the keywords with high frequency were *perineural invasion expression survival innervation growth factor receptor and nerves* which were the fundamental elements of nervous system-tumors in the previous relevant studies. The blue cluster contained keywords such as *cells growth glioblastoma proliferation neurogenesis* and *neurons.* This topic may be about neuronal activity and central nervous system tumors reflecting tumors that promote growth and invasion by altering neural mechanisms. In the yellow cluster we can see the keywords including *activation mechanisms neuropathic pain sensory neurons* and *chemotherapy* suggesting that the mechanism of cancer pain and the effect of chemotherapy on the nervous system have been widely concerned. The green cluster contained *cancer tumor microenvironment breast cancer metastasis stress beta-blockers* and *t-cells.* Keywords in this cluster focused on interactions between the nervous system and various extra-central system cancers as well as stimuli and drug management.

Figure 6B shown the timeline of keywords in cancer neuroscience in the last 5 years. The tree-ring nodes in Figure 6B denote the keywords, and we can see that the size and color of the tree-ring nodes are related to when the keywords occur. The results of the clustering timelines also highlighted key research topics in the field, including *tumor microenvironment, autonomic nerve system | perineural invasion, neuropathic pain, neural-immune interactions, sympathetic nervous system, neurotransmitter receptors, expression,* and *hcn channels.*

We also conducted a burst analysis on keywords, which can provide us with research hotspots and uncover potential future trends in a particular field. Figure 6C displayed the top 10 burst keywords: *vagus nerve* (2.67), *mouse model* (2.25), and *neurotrophic factor* (2.07) were the bursts with the highest strength. Additionally, *neurotrophic factors* were prominent keywords from 2022 to 2024, suggesting that how neurotrophic factors exert their effects between certain nerve cells and tumor cells may be a recent research hotspot.

## 4 Discussion

### 4.1 General information

In this work, we used bibliometric analysis to examine the articles in cancer neuroscience, providing a broad, intuitive grasp of the topic and highlighting current trends. According to our research, there has been an increased trend in this field’s publications during the past 5 years.

This field has gained a great deal of attention since 2019. As the most publications and citations nation in the world, the United States is evident in this field, demonstrating its enormous influence. Subsequent investigation revealed that while Japan and Germany produced fewer publications than China, their articles received far more citations than those published in China. This suggests that China should support creative thinking and innovative research.

Among the top10 journals for publications, many researchers preferred to submit their work to *Cancers*, although *Nature* received a higher citation count. Two noteworthy contributors are Hondermarck H and Monje M. Hondermarck H who joined this field of research early and have been working on how the nervous system affects various cancer progressions since 2012, including nerve fibers, nerve growth factor, neurotransmitters, and their receptors (Li et al., 2022; Pundavela et al., 2015; Magnon and Hondermarck, 2023). Monje M has been devoted to neuronal activity in the microenvironment of gliomas, suggesting for the first time that neurons and gliomas can communicate via paracrine, synaptic electrical signals, providing a profound impact on the development of the discipline (Venkatesh et al., 2015, 2019; Johung and Monje, 2017).

### 4.2 Research focus and hotspots

**Figure 7:**
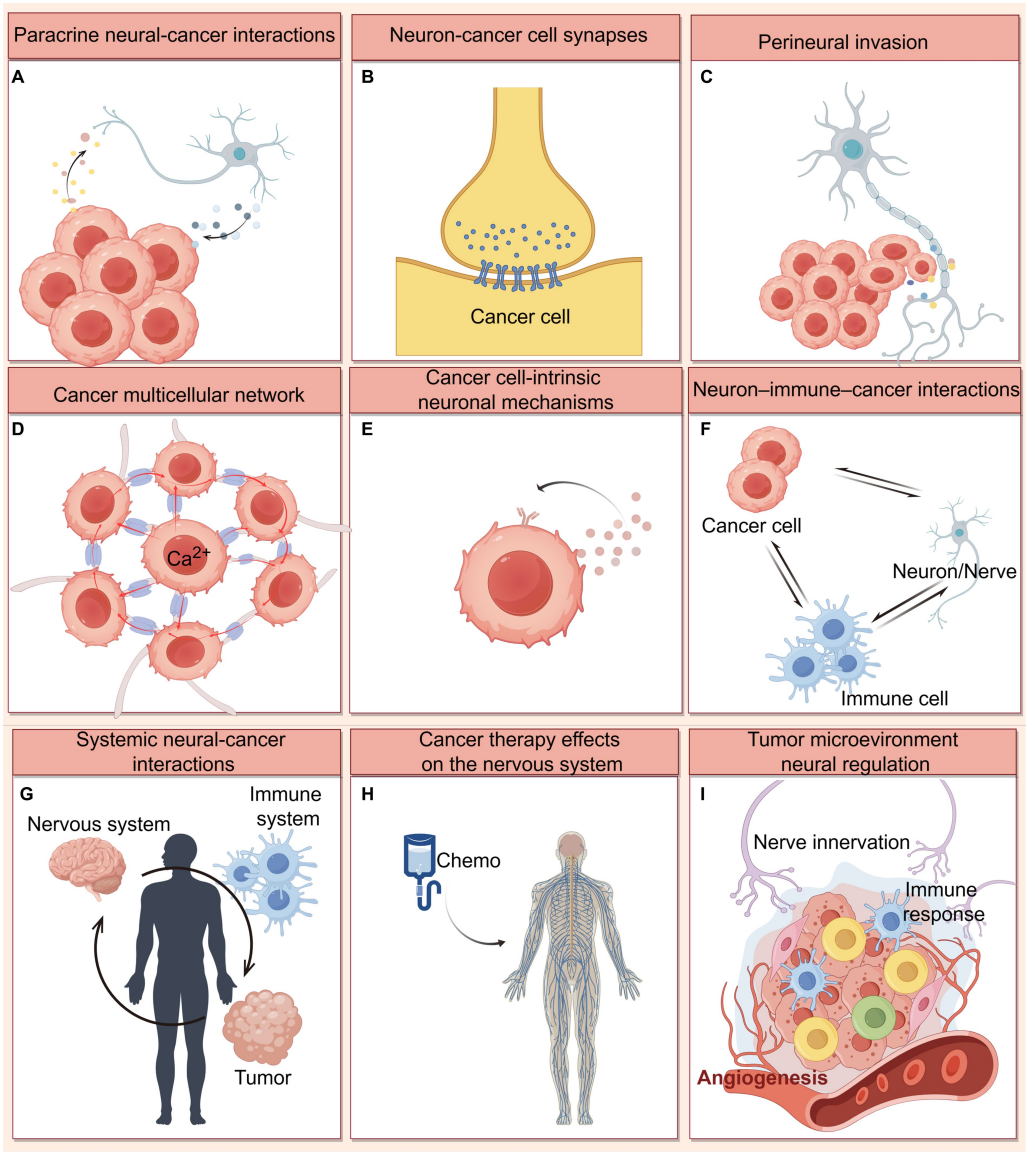
Mechanisms of nervous system-cancer interactions. **(A)** Paracrine signaling between nerve cells and cancer cells, including neuronal activity-dependent paracrine mediators (NLGN3, IGF-1, and BDNF), as well as synaptogenic or axonogenic factors secreted by cancer cells. **(B)** Electrochemical signaling between neurons and brain cancer cells. Direct synaptic signaling mediated by AMPA and GABAA receptors, and indirect synaptic signaling mediated by NMDA receptors on breast cancer brain metastatic cells have been identified. **(C)** Perineural invasion refers to tumor cell growth and invasion along the neural fibers, which involving multiple neural signaling (including neurotransmitter, neurotrophin, and chemokines) in the PNS tumor microenvironment. **(D)** Cancer cells themselves are coupled by gap junctions that communicate via Ca2+ transients, and can achieve the integration of multicellular networks and efficient cellular communication by hijacking neurodevelopmental pathways (connected by TMs and TM-located gap junctions). **(E)** Cancer cells may leverage cell-intrinsic signaling and other processes classically associated with neural cells. For example, cancer cells have been shown to autocrine neurotrophin. **(F)** Extensive cross-talk occurs between neurons/nerves, immune cells and cancer cells mediated by neurotransmitters, neuropeptides. These interactions can influence anti-cancer immunity and pro-cancer inflammation. **(G)** Systemic nervous system-cancer interactions. Cancers can influence nervous system function (like sleep) at a distance through circulating factors, while the nervous system can influence cancer progression through circulating factors such as catecholamines or through altered immune system function. **(H)** Cancer therapies (chemotherapy) frequently cause nervous system toxicities, including impaired function of various types of peripheral nerves and impaired cognitive function. **(I)** Nerve innervation influences on angiogenesis and immune response in the tumor microenvironment. This figure was created by Figdraw (https://www.figdraw.com).

Co-occurrence, timeline, and bursts analysis of keywords indicated that research on cancer neuroscience is primarily concerned with nervous system-tumor interactions, neurons/nerves-immune-cancer crosstalk, as well as the nervous system impairment caused by cancer and cancer therapies. Details can be found in Figure 7.

#### 4.2.1 CNS cancer neuroscience

In the central nervous system (CNS), neuronal-activity promotes glioma progression through paracrine and electrochemical signaling. Paracrine mediators include the neurotrophin brain-derived neurotrophic factor (BDNF), the postsynaptic adhesion protein neuroligin-3 (NLGN3), and insulin-like growth factor 1 (IGF1) (Venkatesh et al., 2015; Chen et al., 2022). Membrane depolarization induced by electrochemical current generated by synaptic signaling is a key mechanism promoting glioma proliferation, like synaptic signaling between glutamatergic neurons and glioma cells via calcium-permeable AMPA receptors (Venkatesh et al., 2019); and synaptic signaling between GABAergic interneurons and diffuse midline glioma cells expressing GABA_A_ receptors (Barron et al., 2023). Additionally, a study identified an indirect peri-synaptic contact: breast cancer brain metastatic cells communicate with glutamatergic neurons via pseudo tripartite synapses (Kamiya et al., 2021). Through these peri-synaptic structures, as well as NMDA receptors on breast cancer cells, glutamatergic signaling promotes tumor growth (Kamiya et al., 2021).

Glioma cells themselves are coupled by gap junctions that communicate via Ca^2+^ transients. Additionally, non-synaptic activity-dependent potassium currents, triggered by neuronal activity, can be enhanced by gap junction-mediated interconnections between glioma cells, resulting in the formation of an electrically coupled network. Consequently, gliomas increase neuronal excitability through this network and positive feedback mechanisms, thus regulating glioma growth (Venkatesh et al., 2015, 2019; Venkataramani et al., 2019). Moreover, tumors can hijack neuro-developmental mechanisms to form ultra-long membranous tube junctions (or tumor microtubules, TMs), which couple cells with gap junction-associated intercellular Ca^2+^ wave signaling and integrate to form tumor cell networks, thereby enhancing the communication efficiency between cancer cells and the resistance to tumor therapy (Latario et al., 2020; Weil et al., 2017). Notably, the latest study found a small, plastic population of extremely active glioblastoma cells that exhibit rhythmic Ca^2+^ oscillations and are particularly connected to others, often at the hub of the network (Hausmann et al., 2023). Hub cells are critical for tumor growth, they can autonomously generate currents that travel through tumor networks connected by gap junctions, driving a tumor-intrinsic periodic depolarization rhythm and subsequent calcium transients (Hausmann et al., 2023).

Neural-cancer interactions are bidirectional. Brain tumors can also induce modifications to the neuronal environment. For example, glutamate released by primary brain tumors causes neuronal hyperexcitability via the xc-cystine-glutamate transporter system (Buckingham et al., 2011). This is believed to be a factor in seizures linked to gliomas and to increase the tumor-promoting effects of neuronal activity (Campbell et al., 2012). Furthermore, glioblastoma remodels human neural circuits by secreting synaptogenic factors (such as thrombospondin-1 and glypican-3), which promote functional tumor and neuronal connectivity while reducing patient survival (Krishna et al., 2023; Lin et al., 2017). Taken together, these evidences suggest that cancer may stimulate neuronal activity in the tumor microenvironment and alter functional neural circuits in the human brain. These processes of neuronal remodeling can further accelerate the development of gliomas by means of paracrine signaling and synaptic electrochemical connections, as discussed in the preceding section.

#### 4.2.2 PNS cancer neuroscience

The peripheral nervous system (PNS) is also believed to be a driver of systemic cancer initiation and progression. Many cancers progression can be modulated by peripheral neurotransmitter and growth factor signaling, including breast cancer, prostate cancer, pancreatic cancer, and skin cancers (Magnon et al., 2013; Kamiya et al., 2019; Hu et al., 2022; Miyato et al., 2011; Renz et al., 2018; Hayakawa et al., 2017; Han et al., 2022; March et al., 2020; Schmitd et al., 2022; Mendenhall et al., 2007). Moreover, the poor prognosis and high recurrence rate of multiple solid tumors have also been shown to be associated with high intra-tumoral nerve density (Ali et al., 2022).

Neurotransmitter-dependent signaling cascades are frequently used to facilitate communication between malignant cells and sympathetic, parasympathetic, or sensory neurons in the tumor microenvironment (Monje et al., 2020). It is necessary to comprehend the function of a particular nerve type in a context-specific manner: intra-tumoral sympathetic nerves facilitate cancer progression via adrenergic signaling (Cole and Sood, 2012; Xia et al., 2019); intra-tumoral parasympathetic nerves may have opposite roles in different tumor types progression via the muscarinic cholinergic receptor, promoting cancer in prostate cancer (Ventura and Evans, 2013), gastric cancer (Gao and Liu, 2021) and colorectal cancer (Zhou et al., 2018), while inhibiting cancer in breast cancer and pancreatic cancer (Hu et al., 2022; Renz et al., 2018). In addition, recent studies have shown that cancer cells outside the CNS can promote axonogenesis by expressing and releasing nerve growth factor, neurotrophic factor, or ephrin B1 (an axon-guidance molecules); they can also recruit new nerve fibers and increase the innervation of the local tumor microenvironment (Ayala et al., 2008; Hayakawa et al., 2017). Beyond axonogenesis in the tumor microenvironment, neurogenesis can also be observed: doublecortin-expressing neural progenitor cells metastasized from the CNS to prostate cancer, where they generated new adrenergic neurons to initiate neurogenesis (Mauffrey et al., 2019), and their density is strongly associated with prostate cancer aggressiveness and recurrence. Similarly, newly formed sympathetic nerves can be detected within breast cancer, which established a polysynaptic connection to the brain, then activated corticotropin-releasing hormone (CRH) neurons in the central medial amygdala (CeM) and promoted tumor growth through CeM^CRH^ neurons and the CeM^CRH^ → lateral paragigantocellular nucleus (LPGi) circuit (Xiong et al., 2023).

Importantly, cancer progression can be profoundly influenced by cross-talk among immune cells, tumor cells, and neurons or neuronal. Infiltrating nerves not only affect the tumor cells but also other cells inside the tumor microenvironment (Zahalka and Frenette, 2020). For instance, it has been demonstrated that the immunosuppressive potential of myeloid suppressor cells in the tumor microenvironment can be modulated by β2-adrenergic signaling (Mohammadpour et al., 2019); it has also been demonstrated that the parasympathetic nerves modulate cytotoxic T cells in the spleen, which in turn contributes to colorectal carcinogenesis (Dubeykovskaya et al., 2016). Notably, B lymphocytes have been found to reduce CD^8+^ T lymphocytes function via the autocrine neurotransmitter GABA signaling; GABA secreted by B lymphocytes also induces tumor-associated macrophages to develop an immune-suppressive phenotype, thereby preventing anti-cancer immunity and promoting tumor growth (Zhang et al., 2021). Furthermore, on a systemic level, mammary gland tumors exhibited abnormal relationships between immunity, metabolism, and sleep. Tumor-bearing mice exhibited interleukin-6 (IL-6)-mediated peripheral inflammation, which coincided with disrupting metabolism and sleep by altering specific neuronal populations (Walker et al., 2019). While during acute stress, leukocyte distribution and function were shaped in distinct brain regions, corticotropin-releasing hormone neuron-mediated leukocyte shifts protected against the acquisition of autoimmunity, hence calibrating the immune system’s response to physical threats (Poller et al., 2022). In conclusion, neurons, immune cells, and cancer cells are forming a triangular relationship. These discoveries underscore the importance of the neuro-immune-tumor crosstalk for tumor progression and may provide us with targets for immunotherapy against tumor progression, despite the fact that there are still many questions to be explored in cancer neuroscience.

#### 4.2.3 Cancer therapies and neurological impairment

It is well known that conventional chemotherapy and radiotherapy can cause neurotoxicity in cancer patients (Stone and DeAngelis, 2016), and trigger cognitive symptoms of debilitating syndromes (Gibson et al., 2019; Pease-Raissi et al., 2017), as evidenced by deficits in memory, attention, processing speed, multitasking, and executive function, as well as neuropathies of sensory, motor, and autonomic nerves (Wefel et al., 2004; Staff et al., 2017).

Cancer patients receiving chemotherapy have been demonstrated that not only experience a reduction in grey matter volume/density and white matter integrity, but also an alteration in grey and white matter structural network connectivity. These abnormalities are most pronounced in frontal and temporal brain regions (McDonald, 2021). The use of chemotherapeutic agents has also been found to damage hippocampal synapses and dendrites: for example, treatment with clinically relevant doses of cisplatin leads to a reduction of dendritic branches and decreased spine density in CA1 and CA3 hippocampal neurons, which correlates with the degree of neuronal connectivity and function (Andres et al., 2014). In addition to chemotherapy-induced neural impairment, cranial radiation therapy also leads to debilitating cognitive decline, characterized by hippocampal dysfunction and persistent radiation-induced microglial inflammation (Monje et al., 2007), which is associated with defects in the proliferative capacity of neural progenitor cells, synaptic plasticity-associated cytoskeletal signaling pathways, and reduced hippocampal neurogenesis (Monje et al., 2002; Parihar and Limoli, 2013; Kempf et al., 2014).

In summary, the multiple mechanisms of tumor-neural crosstalk and its associated clinicopathological consequences, as well as the mechanisms and implications of cancer treatment-induced neurotoxicity, urge us to develop optimal therapeutic strategies to effectively treat cancer and reduce debilitating neurological side effects. Currently, various clinical trials are being inspired to develop more effective anti-cancer therapies. For example, neuromodulating drugs (such as beta-adrenergic, dopamine, and glutamate receptor modulators) are designed to target neurotransmitters or neuropeptides that promote tumor growth (Melhem-Bertrandt et al., 2011; Udumyan et al., 2017; Caudill et al., 2011).

## 5 Limitations

This study provides a pioneering attempt to use bibliometrics to investigate research trends and hot spots in cancer neuroscience. However, we must acknowledge some limitations of this study.

Firstly, in order to ensure the norms and standards of bibliometric analysis, articles in the WoSCC database were selected for this study, excluding other databases. This will unavoidably result in inadequate data analysis, even though this database is the most prestigious collection of scientific papers, guaranteeing the high quality of the collected data.

Furthermore, bibliometrics evaluates the quality of time-related research. Although cancer neuroscience is still a developing field and only publications from 2020 to 2024 can be used, our bibliometric study can provide key trends in the field and make predictions about future trends. By investigating current research trends and emerging interests, this study provides valuable insights into understanding the crosstalk mechanisms between different types of cancer and the nervous system during cancer development.

## 6 Conclusion

Through our comprehensive bibliometric analysis, we see a growing interest in the field among researchers and scholars. Perineural invasion, innervation, growth factor, receptor, glioblastoma, neurogenesis, neuropathic pain, chemotherapy, tumor microenvironment, breast cancer, stress, beta-blockers, and T cells. These are all the main focus and hot spots in the field. Researchers and scholars have made significant progress in elucidating nervous system-tumor interactions (Mancusi and Monje, 2023; Hanahan and Monje, 2023; Winkler et al., 2023; Shi et al., 2022). However, there are still many unclear questions in this field, like the differences of the nervous system interacts with CNS tumors or tumors outside the CNS, the mechanisms and effects of the perineuronal niche on the composition and evolution of the microenvironment during tumor development. Further research would require cross-disciplinary research thinking, applying knowledge from fields such as neuroscience, cancer biology, immunology and developmental biology. Another key requirement for the future will be to map the nervous system-cancer crosstalk more thoroughly on a variety of scales and levels, which helps us to decipher biomarkers targeting nervous system–cancer interactions. Biomarkers and cancer vaccines will be new directions for the development of cancer neuroscience. Clinical drug modulating these key factors (such as neuropeptides, neurotransmitter receptors, ion channels), in combination with surgery, radiotherapy and chemotherapy as well as immunotherapies, holds great potential in interrupting tumor growth, spread and treatment resistance. All of these insights are essential for developing a more thorough grasp of the field of cancer neuroscience and for directing cancer treatments.

## Data Availability

The original contributions presented in the study are included in the article/supplementary material, further inquiries can be directed to the corresponding authors.

## References

[ref1] Ahmad P. Slots J. (2021). A bibliometric analysis of periodontology. Periodontol. 2000 85, 237–240. doi: 10.1111/prd.12376 33226679

[ref2] Ali S. R. Jordan M. Nagarajan P. Amit M. (2022). Nerve density and neuronal biomarkers in Cancer. Cancers 14:4817. doi: 10.3390/cancers14194817, PMID: 36230740 PMC9561962

[ref3] Andres A. L. Gong X. Di K. Bota D. A. (2014). Low-doses of cisplatin injure hippocampal synapses: a mechanism for “chemo” brain? Exp. Neurol. 255, 137–144. doi: 10.1016/j.expneurol.2014.02.020, PMID: 24594220 PMC4059602

[ref4] Ayala G. E. Dai H. Powell M. Li R. Ding Y. Wheeler T. M.. (2008). Cancer-related axonogenesis and neurogenesis in prostate cancer. Clin. Cancer Res. 14, 7593–7603. doi: 10.1158/1078-0432.CCR-08-1164, PMID: 19047084

[ref5] Barron T. Yalçin B. Mochizuki A. Cantor E. Shamardani K. Tlais D.. (2023). GABAERGIC neuron-to-glioma synapses in diffuse midline gliomas. Neuro-Oncology 25:i11. doi: 10.1093/neuonc/noad073.044 PMC11946904 39972132

[ref6] Boilly B. Faulkner S. Jobling P. Hondermarck H. (2017). Nerve dependence: from regeneration to Cancer. Cancer Cell 31, 342–354. doi: 10.1016/j.ccell.2017.02.005 28292437

[ref7] Buckingham S. C. Campbell S. L. Haas B. R. Montana V. Robel S. Ogunrinu T.. (2011). Glutamate release by primary brain tumors induces epileptic activity. Nat. Med. 17, 1269–1274. doi: 10.1038/nm.2453, PMID: 21909104 PMC3192231

[ref8] Campbell S. L. Buckingham S. C. Sontheimer H. (2012). Human glioma cells induce hyperexcitability in cortical networks. Epilepsia 53, 1360–1370. doi: 10.1111/j.1528-1167.2012.03557.x, PMID: 22709330 PMC3418468

[ref9] Caudill J. S. Brown P. D. Cerhan J. H. Rummans T. A. (2011). Selective serotonin reuptake inhibitors, glioblastoma multiforme, and impact on toxicities and overall survival the mayo clinic experience. Am. J. Clin. Oncol. 34, 385–387. doi: 10.1097/COC.0b013e3181e8461a 20859197

[ref10] Chen R. Liu Z. Wang J. Jin W. Abdu H. I. Pei J.. (2022). A review of the nutritional value and biological activities of sturgeon processed byproducts. Front. Nutr. 9:1024309. doi: 10.3389/fnut.2022.1024309, PMID: 36451740 PMC9702528

[ref11] Chen P. X. Wang W. Liu R. Lyu J. H. Zhang L. Li B. Z.. (2022). Olfactory sensory experience regulates gliomagenesis via neuronal IGF1. Nature 606, 550–556. doi: 10.1038/s41586-022-04719-9, PMID: 35545672

[ref12] Cole S. W. Sood A. K. (2012). Molecular pathways: beta-adrenergic signaling in cancer. Clin. Cancer Res. 18, 1201–1206. doi: 10.1158/1078-0432.CCR-11-0641, PMID: 22186256 PMC3294063

[ref13] Cui Q. Jiang D. Zhang Y. Chen C. (2023). The tumor-nerve circuit in breast cancer. Cancer Metastasis Rev. 42, 543–574. doi: 10.1007/s10555-023-10095-1, PMID: 36997828 PMC10349033

[ref14] Dubeykovskaya Z. Si Y. L. Chen X. W. Worthley D. L. Renz B. W. Urbanska A. M.. (2016). Neural innervation stimulates splenic TFF2 to arrest myeloid cell expansion and cancer. Nat. Commun. 7:10517. doi: 10.1038/ncomms10517 26841680 PMC4742920

[ref15] Faulkner S. Jobling P. March B. Jiang C. C. Hondermarck H. (2019). Tumor neurobiology and the war of nerves in Cancer. Cancer Discov. 9, 702–710. doi: 10.1158/2159-8290.CD-18-1398, PMID: 30944117

[ref16] Gao J. Liu S.-G. (2021). Role of sympathetic and parasympathetic nerves in the development of gastric cancer through antagonism. Chin. Med. J. 134, 908–909. doi: 10.1097/CM9.0000000000001348, PMID: 33470652 PMC8078313

[ref17] Gibson E. M. Nagaraja S. Ocampo A. Tam L. T. Wood L. S. Pallegar P. N.. (2019). Methotrexate chemotherapy induces persistent tri-glial dysregulation that underlies chemotherapy-related cognitive impairment. Cell 176:43. doi: 10.1016/j.cell.2018.10.049 30528430 PMC6329664

[ref18] Han S. Wang D. Huang Y. Zeng Z. Xu P. Xiong H.. (2022). A reciprocal feedback between colon cancer cells and Schwann cells promotes the proliferation and metastasis of colon cancer. J. Exp. Clin. Cancer Res. 41:348. doi: 10.1186/s13046-022-02556-2, PMID: 36522730 PMC9753336

[ref19] Hanahan D. (2022). Hallmarks of Cancer: new dimensions. Cancer Discov. 12, 31–46. doi: 10.1158/2159-8290.CD-21-1059, PMID: 35022204

[ref20] Hanahan D. Monje M. (2023). Cancer hallmarks intersect with neuroscience in the tumor microenvironment. Cancer Cell 41, 573–580. doi: 10.1016/j.ccell.2023.02.012, PMID: 36917953 PMC10202656

[ref21] Hassan W. Zafar M. Duarte A. E. Kamdem J. P. Teixeira da Rocha J. B. (2021). Pharmacological research: a bibliometric analysis from 1989 to 2019. Pharmacol. Res. 169:105645. doi: 10.1016/j.phrs.2021.105645 33957268

[ref22] Hausmann D. Hoffmann D. C. Venkataramani V. Jung E. Horschitz S. Tetzlaff S. K.. (2023). Autonomous rhythmic activity in glioma networks drives brain tumour growth. Nature 613, 179–186. doi: 10.1038/s41586-022-05520-4, PMID: 36517594

[ref23] Hayakawa Y. Sakitani K. Konishi M. Asfaha S. Niikura R. Tomita H.. (2017). Nerve growth factor promotes gastric tumorigenesis through aberrant cholinergic signaling. Cancer Cell 31, 21–34. doi: 10.1016/j.ccell.2016.11.005, PMID: 27989802 PMC5225031

[ref24] Hu J. M. Chen W. Z. Shen L. S. Chen Z. G. Huang J. (2022). Crosstalk between the peripheral nervous system and breast cancer influences tumor progression. Biochimica et Biophysica Acta-reviews on. Cancer 1877:188828. doi: 10.1016/j.bbcan.2022.188828 36283598

[ref25] Jiang S. Liu Y. Zheng H. Zhang L. Zhao H. Sang X.. (2023). Evolutionary patterns and research frontiers in neoadjuvant immunotherapy: a bibliometric analysis. Int. J. Surg. 109, 2774–2783. doi: 10.1097/JS9.0000000000000492, PMID: 37216225 PMC10498839

[ref26] Johung T. Monje M. (2017). Neuronal activity in the glioma microenvironment. Curr. Opin. Neurobiol. 47, 156–161. doi: 10.1016/j.conb.2017.10.009, PMID: 29096244 PMC5927594

[ref27] Kamiya A. Hayama Y. Kato S. Shimomura A. Shimomura T. Irie K.. (2019). Genetic manipulation of autonomic nerve fiber innervation and activity and its effect on breast cancer progression. Nat. Neurosci. 22, 1289–1305. doi: 10.1038/s41593-019-0430-3, PMID: 31285612

[ref28] Kamiya A. Hiyama T. Fujimura A. Yoshikawa S. (2021). Sympathetic and parasympathetic innervation in cancer: therapeutic implications. Clin. Auton. Res. 31, 165–178. doi: 10.1007/s10286-020-00724-y, PMID: 32926324

[ref29] Kempf S. J. Buratovic S. von Toerne C. Moertl S. Stenerlöw B. Hauck S. M.. (2014). Ionising radiation immediately impairs synaptic plasticity-associated cytoskeletal signalling pathways in HT22 cells and in mouse brain: an in vitro/in vivo comparison study. PLoS One 9:e110464. doi: 10.1371/journal.pone.0110464, PMID: 25329592 PMC4203799

[ref30] Krishna S. Choudhury A. Keough M. B. Seo K. Ni L. Kakaizada S.. (2023). Glioblastoma remodelling of human neural circuits decreases survival. Nature 617, 599–607. doi: 10.1038/s41586-023-06036-1, PMID: 37138086 PMC10191851

[ref31] Latario C. J. Schoenfeld L. W. Howarth C. L. Pickrell L. E. Begum F. Fischer D. A.. (2020). Tumor microtubes connect pancreatic cancer cells in an Arp2/3 complex-dependent manner. Mol. Biol. Cell 31, 1259–1272. doi: 10.1091/mbc.E19-11-0605, PMID: 32267199 PMC7353147

[ref32] Le C. P. Nowell C. J. Kim-Fuchs C. Botteri E. Hiller J. G. Ismail H.. (2016). Chronic stress in mice remodels lymph vasculature to promote tumour cell dissemination. Nat. Commun. 7:7. doi: 10.1038/ncomms10634 PMC4773495 26925549

[ref33] Li D. Hu L. N. Zheng S. M. La T. Wei L. Y. Zhang X. J.. (2022). High nerve density in breast cancer is associated with poor patient outcome. FASEB Bioadv. 4, 391–401. doi: 10.1096/fba.2021-00147, PMID: 35664834 PMC9164247

[ref34] Lin C. C. J. Yu K. Hatcher A. Huang T. W. Lee H. K. Carlson J.. (2017). Identification of diverse astrocyte populations and their malignant analogs. Nat. Neurosci. 20, 396–405. doi: 10.1038/nn.4493 28166219 PMC5824716

[ref35] Liu Y. Cheng X. Han X. Cheng X. Jiang S. Lin Y.. (2022). Global research landscape and trends of lung cancer immunotherapy: a bibliometric analysis. Front. Immunol. 13:1032747. doi: 10.3389/fimmu.2022.1032747, PMID: 36532038 PMC9751816

[ref36] Liu X. Zhao S. Tan L. Tan Y. Wang Y. Ye Z.. (2022). Frontier and hot topics in electrochemiluminescence sensing technology based on CiteSpace bibliometric analysis. Biosens. Bioelectron. 201:113932. doi: 10.1016/j.bios.2021.113932, PMID: 35065388

[ref37] Magnon C. Hall S. J. Lin J. Xue X. Gerber L. Freedland S. J.. (2013). Autonomic nerve development contributes to prostate cancer progression. Science 341:1236361. doi: 10.1126/science.1236361, PMID: 23846904

[ref38] Magnon C. Hondermarck H. (2023). The neural addiction of cancer. Nat. Rev. Cancer 23, 317–334. doi: 10.1038/s41568-023-00556-8 37041409

[ref39] Mancusi R. Monje M. (2023). The neuroscience of cancer. Nature 618, 467–479. doi: 10.1038/s41586-023-05968-y, PMID: 37316719 PMC11146751

[ref40] March B. Faulkner S. Jobling P. Steigler A. Blatt A. Denham J.. (2020). Tumour innervation and neurosignalling in prostate cancer. Nat. Rev. Urol. 17, 119–130. doi: 10.1038/s41585-019-0274-3, PMID: 31937919

[ref41] Mauffrey P. Tchitchek N. Barroca V. Bemelmans A. Firlej V. Allory Y.. (2019). Progenitors from the central nervous system drive neurogenesis in cancer. Nature 569, 672–678. doi: 10.1038/s41586-019-1219-y 31092925

[ref42] McDonald B. C. (2021). Structural neuroimaging findings related to adult non-CNS Cancer and treatment: review, integration, and implications for treatment of cognitive dysfunction. Neurotherapeutics 18, 792–810. doi: 10.1007/s13311-021-01096-5, PMID: 34402034 PMC8423886

[ref43] Melhem-Bertrandt A. Chavez-MacGregor M. Lei X. D. Brown E. N. Lee R. T. Meric-Bernstam F.. (2011). Beta-blocker use is associated with improved relapse-free survival in patients with triple-negative breast Cancer. J. Clin. Oncol. 29, 2645–2652. doi: 10.1200/JCO.2010.33.4441, PMID: 21632501 PMC3139371

[ref44] Mendenhall W. M. Amdur R. J. Hinerman R. W. Werning J. W. Malyapa R. S. Villaret D. B.. (2007). Skin cancer of the head and neck with perineural invasion. Am. J. Clin. Oncol. 30, 93–96. doi: 10.1097/01.coc.0000251224.16075.60 17278901

[ref45] Miyato H. Kitayama J. Ishigami H. Kaisaki S. Nagawa H. (2011). Loss of sympathetic nerve fibers around intratumoral arterioles reflects malignant potential of gastric cancer. Ann. Surg. Oncol. 18, 2281–2288. doi: 10.1245/s10434-011-1562-1, PMID: 21290194

[ref46] Mohammadpour H. MacDonald C. R. Qiao G. X. Chen M. H. Dong B. W. Hylander B. L.. (2019). β2 adrenergic receptor-mediated signaling regulates the immunosuppressive potential of myeloid-derived suppressor cells. J. Clin. Invest. 129, 5537–5552. doi: 10.1172/JCI129502, PMID: 31566578 PMC6877316

[ref47] Monje M. Borniger J. C. D'Silva N. J. Deneen B. Dirks P. B. Fattahi F.. (2020). Roadmap for the emerging field of Cancer neuroscience. Cell 181, 219–222. doi: 10.1016/j.cell.2020.03.034, PMID: 32302564 PMC7286095

[ref48] Monje M. L. Mizumatsu S. Fike J. R. Palmer T. D. (2002). Irradiation induces neural precursor-cell dysfunction. Nat. Med. 8, 955–962. doi: 10.1038/nm749, PMID: 12161748

[ref49] Monje M. L. Vogel H. Masek M. Ligon K. L. Fisher P. G. Palmer T. D. (2007). Impaired human hippocampal neurogenesis after treatment for central nervous system malignancies. Ann. Neurol. 62, 515–520. doi: 10.1002/ana.21214, PMID: 17786983

[ref50] Ninkov A. Frank J. R. Maggio L. A. (2022). Bibliometrics: methods for studying academic publishing. Perspect. Med. Educ. 11, 173–176. doi: 10.1007/s40037-021-00695-4, PMID: 34914027 PMC9240160

[ref51] Pan C. Winkler F. (2022). Insights and opportunities at the crossroads of cancer and neuroscience. Nat. Cell Biol. 24, 1454–1460. doi: 10.1038/s41556-022-00978-w, PMID: 36097070

[ref52] Parihar V. K. Limoli C. L. (2013). Cranial irradiation compromises neuronal architecture in the hippocampus. Proc. Natl. Acad. Sci. USA 110, 12822–12827. doi: 10.1073/pnas.1307301110, PMID: 23858442 PMC3732939

[ref53] Pease-Raissi S. E. Pazyra-Murphy M. F. Li Y. Wachter F. Fukuda Y. Fenstermacher S. J.. (2017). Paclitaxel reduces axonal Bclw to initiate IP3R1-dependent axon degeneration. Neuron 96, 373–386.e6. doi: 10.1016/j.neuron.2017.09.034, PMID: 29024661 PMC5680044

[ref54] Poller W. C. Downey J. Mooslechner A. A. Khan N. Li L. Chan C. T.. (2022). Brain motor and fear circuits regulate leukocytes during acute stress. Nature 607, 578–584. doi: 10.1038/s41586-022-04890-z 35636458 PMC9798885

[ref55] Priovashini C. Mallick B. (2022). A bibliometric review on the drivers of environmental migration. Ambio 51, 241–252. doi: 10.1007/s13280-021-01543-9, PMID: 33738730 PMC8651838

[ref56] Pundavela J. Roselli S. Faulkner S. Attia J. Scott R. J. Thorne R. F.. (2015). Nerve fibers infiltrate the tumor microenvironment and are associated with nerve growth factor production and lymph node invasion in breast cancer. Mol. Oncol. 9, 1626–1635. doi: 10.1016/j.molonc.2015.05.001, PMID: 26009480 PMC5528785

[ref57] Renz B. W. Takahashi R. Tanaka T. Macchini M. Hayakawa Y. Dantes Z.. (2018). β2 adrenergic-Neurotrophin feedforward loop promotes pancreatic Cancer. Cancer Cell 33, 75–90.e7. doi: 10.1016/j.ccell.2017.11.007, PMID: 29249692 PMC5760435

[ref58] Renz B. W. Tanaka T. Sunagawa M. Takahashi R. Jiang Z. Macchini M.. (2018). Cholinergic signaling via muscarinic receptors directly and indirectly suppresses pancreatic tumorigenesis and Cancer Stemness. Cancer Discov. 8, 1458–1473. doi: 10.1158/2159-8290.CD-18-0046, PMID: 30185628 PMC6214763

[ref59] Saloman J. L. Albers K. M. Li D. Hartman D. J. Crawford H. C. Muha E. A.. (2016). Ablation of sensory neurons in a genetic model of pancreatic ductal adenocarcinoma slows initiation and progression of cancer. Proc. Natl. Acad. Sci. USA 113, 3078–3083. doi: 10.1073/pnas.1512603113, PMID: 26929329 PMC4801275

[ref60] Schmitd L. B. Perez-Pacheco C. Bellile E. L. Wu W. Casper K. Mierzwa M.. (2022). Spatial and transcriptomic analysis of Perineural invasion in Oral Cancer. Clin. Cancer Res. 28, 3557–3572. doi: 10.1158/1078-0432.CCR-21-4543, PMID: 35819260 PMC9560986

[ref61] Selvaggi F. Melchiorre E. Casari I. Cinalli S. Cinalli M. Aceto G. M.. (2022). Perineural invasion in pancreatic ductal adenocarcinoma: from molecules towards drugs of clinical relevance. Cancers 14:5793. doi: 10.3390/cancers14235793, PMID: 36497277 PMC9739544

[ref62] Shi D. D. Guo J. A. Hoffman H. I. Su J. Mino-Kenudson M. Barth J. L.. (2022). Therapeutic avenues for cancer neuroscience: translational frontiers and clinical opportunities. Lancet Oncol. 23, e62–e74. doi: 10.1016/S1470-2045(21)00596-9, PMID: 35114133 PMC9516432

[ref63] Staff N. P. Grisold A. Grisold W. Windebank A. J. (2017). Chemotherapy-induced peripheral neuropathy: a current review. Ann. Neurol. 81, 772–781. doi: 10.1002/ana.24951, PMID: 28486769 PMC5656281

[ref64] Stone J. B. DeAngelis L. M. (2016). Cancer-treatment-induced neurotoxicity—focus on newer treatments. Nat. Rev. Clin. Oncol. 13, 92–105. doi: 10.1038/nrclinonc.2015.152 26391778 PMC4979320

[ref65] Udumyan R. Montgomery S. Fang F. Almroth H. Valdimarsdottir U. Ekbom A.. (2017). Beta-blocker drug use and survival among patients with pancreatic adenocarcinoma. Cancer Res. 77, 3700–3707. doi: 10.1158/0008-5472.CAN-17-0108 28473530

[ref66] Venkataramani V. Tanev D. I. Strahle C. Studier-Fischer A. Fankhauser L. Kessler T.. (2019). Glutamatergic synaptic input to glioma cells drives brain tumour progression. Nature 573, 532–538. doi: 10.1038/s41586-019-1564-x, PMID: 31534219

[ref67] Venkatesh H. S. Johung T. B. Caretti V. Noll A. Tang Y. J. Nagaraja S.. (2015). Neuronal activity promotes glioma growth through Neuroligin-3 secretion. Cell 161, 803–816. doi: 10.1016/j.cell.2015.04.012, PMID: 25913192 PMC4447122

[ref68] Venkatesh H. S. Morishita W. Geraghty A. C. Silverbush D. Gillespie S. M. Arzt M.. (2019). Electrical and synaptic integration of glioma into neural circuits. Nature 573, 539–545. doi: 10.1038/s41586-019-1563-y 31534222 PMC7038898

[ref69] Ventura S. Evans B. A. (2013). Does the autonomic nervous system contribute to the initiation and progression of prostate cancer? Asian J. Androl. 15, 715–716. doi: 10.1038/aja.2013.114 24141535 PMC3854038

[ref70] Villers A. McNeal J. E. Redwine E. A. Freiha F. S. Stamey T. A. (1989). The role of perineural space invasion in the local spread of prostatic adenocarcinoma. J. Urol. 142, 763–768. doi: 10.1016/S0022-5347(17)38881-X, PMID: 2769857

[ref71] Walker W. H. Borniger J. C. Nelson R. J. DeVries A. C. (2019). A role for hypocretin/orexin in metabolic and sleep abnormalities in a mouse model of non-metastatic breast cancer. Brain Behav. Immun. 76:e19. doi: 10.1016/j.bbi.2018.11.231 PMC6031468 29805100

[ref72] Wefel J. S. Kayl A. E. Meyers C. A. (2004). Neuropsychological dysfunction associated with cancer and cancer therapies: a conceptual review of an emerging target. Br. J. Cancer 90, 1691–1696. doi: 10.1038/sj.bjc.6601772, PMID: 15150608 PMC2410277

[ref73] Wei J. Su W. Zhao Y. Wei Z. Hua Y. Xue P.. (2022). Maresin 1 promotes nerve regeneration and alleviates neuropathic pain after nerve injury. J. Neuroinflammation 19:32. doi: 10.1186/s12974-022-02405-1, PMID: 35109876 PMC8809034

[ref74] Wei N. Xu Y. Yn L. Shi J. Zhang X. You Y.. (2022). A bibliometric analysis of T cell and atherosclerosis. Front. Immunol. 13:948314. doi: 10.3389/fimmu.2022.948314, PMID: 36311729 PMC9606647

[ref75] Weil S. Osswald M. Solecki G. Grosch J. Jung E. Lemke D.. (2017). Tumor microtubes convey resistance to surgical lesions and chemotherapy in gliomas. Neuro-Oncology 19, 1316–1326. doi: 10.1093/neuonc/nox070 28419303 PMC5596180

[ref76] Winkler F. Venkatesh H. S. Amit M. Batchelor T. Demir I. E. Deneen B.. (2023). Cancer neuroscience: state of the field, emerging directions. Cell 186, 1689–1707. doi: 10.1016/j.cell.2023.02.002, PMID: 37059069 PMC10107403

[ref77] Wu F. Gao J. Kang J. Wang X. Niu Q. Liu J.. (2022). Knowledge mapping of exosomes in autoimmune diseases: a bibliometric analysis (2002–2021). Front. Immunol. 13:939433. doi: 10.3389/fimmu.2022.939433, PMID: 35935932 PMC9353180

[ref78] Xia Y. Wei Y. Li Z.-Y. Cai X.-Y. Zhang L.-L. Dong X.-R.. (2019). Catecholamines contribute to the neovascularization of lung cancer via tumor-associated macrophages. Brain Behav. Immun. 81, 111–121. doi: 10.1016/j.bbi.2019.06.004, PMID: 31176001

[ref79] Xiong S.-Y. Wen H.-Z. Dai L.-M. Lou Y.-X. Wang Z.-Q. Yi Y.-L.. (2023). A brain-tumor neural circuit controls breast cancer progression in mice. J. Clin. Invest. 133:e167725. doi: 10.1172/JCI167725, PMID: 37847562 PMC10721160

[ref80] Yeung A. W. K. (2019). Comparison between Scopus, web of science, PubMed and publishers for mislabelled review papers. Curr. Sci. 116:1909. doi: 10.18520/cs/v116/i11/1909-1914

[ref81] Young H. H. (1897). On the presence of nerves in tumors and of other structures in them as revealed by a modification of EHRLICH'S method of "vital staining" with methylene blue. J. Exp. Med. 2, 49–105. doi: 10.1084/jem.2.1.1, PMID: 19866822 PMC2117917

[ref82] Zahalka A. H. Arnal-Estapé A. Maryanovich M. Nakahara F. Cruz C. D. Finley L. W. S.. (2017). Adrenergic nerves activate an angio-metabolic switch in prostate cancer. Science 358, 321–326. doi: 10.1126/science.aah5072 29051371 PMC5783182

[ref83] Zahalka A. H. Frenette P. S. (2020). Nerves in cancer. Nat. Rev. Cancer 20, 143–157. doi: 10.1038/s41568-019-0237-2, PMID: 31974491 PMC7709871

[ref84] Zhang B. H. Vogelzang A. Miyajima M. Sugiura Y. Wu Y. B. Chamoto K.. (2021). B cell-derived GABA elicits IL-10+ macrophages to limit anti-tumour immunity. Nature 599, 471–476. doi: 10.1038/s41586-021-04082-1 34732892 PMC8599023

[ref85] Zhou H. Shi B. Jia Y. Qiu G. Yang W. Li J.. (2018). Expression and significance of autonomic nerves and α9 nicotinic acetylcholine receptor in colorectal cancer. Mol. Med. Rep. 17, 8423–8431. doi: 10.3892/mmr.2018.8883, PMID: 29658602

